# Residential Mobility Decreases Neural Responses to Social Norm Violation

**DOI:** 10.3389/fpsyg.2019.02654

**Published:** 2019-11-28

**Authors:** Siyang Luo, Qianting Kong, Zijun Ke, Yiyi Zhu, Liqin Huang, Meihua Yu, Ying Xu

**Affiliations:** Department of Psychology, Guangdong Key Laboratory of Social Cognitive Neuroscience and Mental Health, Guangdong Provincial Key Laboratory of Brain Function and Disease, Sun Yat-sen University, Guangzhou, China

**Keywords:** residential mobility, social norm violations, event-related potential, mobility, EEG

## Abstract

Social norms are essential, but they vary across cultures and societies. With the internationalization of human society, population mobility has greatly increased, especially in developing countries, which can have an impact on people’s psychological states and behaviors and result in sociocultural change. The current research used three studies to examine the hypothesis that residential mobility plays a crucial role in the perception of social norm violations. Study 1 used an association test and found that residential mobility was correlated with the perception of both weak and strong social norm violations in females. Study 2 combined electroencephalography and found a negative differential N400 between weak social norm violations and appropriate behavior between residentially mobile and stable mindsets, suggesting that residential mobility modulates individuals’ detection of social norm-violating behavior. Study 3 revealed that residential mobility does not have a similar effect on semantic violations, which indicates that the effect of residential mobility does not occur in non-social norm violations. Our findings provide insight into how and why individuals’ detection of social norm-violating behaviors varies according to the dynamic development of society. As residential mobility continues to increase worldwide, especially in developing countries, more attention should be paid to the concomitant impact during the course of sociocultural change to build a better strategy for cultural specific social governance.

## Introduction

In recent decades, countless people have moved to new cities or countries for better jobs or education. Schools and colleges provide students with opportunities for exchange, and corporations offer employees chances to work in other cities. Thus far, residential mobility has been relatively high in North America (Castles et al., 2013). Because of the rapid economic development in Asia and cooperation among countries, residential mobility in this region is expected to increase (Czaika and De Haas, 2014; International Organization for Migration, 2015). Africa, which is described in the literature as an emigrant location, also shows an increased trend in residential mobility because African countries have increased their attractiveness (Jonsson, 2009). Thus, residential mobility appears to be increasing worldwide. The World Migration Report (International Organization for Migration, 2015) revealed that there are currently more than 232 million international migrants and 740 million internal migrants. Moreover, an additional 2.5 billion people are expected to move to urban areas in Asia and Africa.

Nevertheless, residential mobility is associated with several problems. For example, residentially mobile areas have higher crime rates than stable cities (Sampson et al., 1997). However, an extended study conducted by Cohen (1998) revealed that residential mobility was not necessarily related to increased violence, and the association was dependent on the acquiescent culture shared by the community. In communities that valued a culture of honor, less stable regions were related to decreased argument-related homicide but were associated with increased felony-related homicide compared to more stable regions (Cohen, 1998). To extend these research areas, we investigated how residential mobility influences social norm violations that are not formally sanctioned.

Residential mobility is defined as the frequency with which people change residences. At the individual level, this concept refers to the number of residential moves that a person experiences during a specific period or moves that are expected in the future (Oishi, 2010). Although residential moves can be exciting, they can lead to short- and long-term psychological, cognitive and behavioral consequences (Oishi and Talhelm, 2012). For example, high residential mobility may induce anxiety and foster familiarity seeking (Oishi et al., 2012). People who move frequently are motivated to expand social networks because of loneliness (Oishi et al., 2013), and they are more likely to help strangers than are people who are residentially stable (Lun et al., 2012). One primary difference between stable and mobile individuals is the state of their social relationships. Frequent movers tend to have a social network with few responsibilities, whereas those who are residentially stable tend to have stable friendships with obligations and duties (Ho et al., 2006; Oishi, 2010). In stable societies, social ties are deep and friends are obliged to help each other in times of need; occasionally, excessive costs are associated with helping. Thus, in a residentially stable context, people must be cautious of friends and ensure that they are trustworthy. In stable societies, when friends are ill-behaved, people may impose punishments to deter inappropriate behavior. Indeed, research has found that East Asians are more likely to punish inappropriate behavior than North Americans are (Wang and Leung, 2010). Compared with people in mobile societies, people in stable societies may have stronger sensitivities to norm violations to ensure that their friends are trustworthy and avoid punishments associated with deviant behavior. Thus, we hypothesized that residential mobility would decrease the detection of social norm violations.

A norm can be a pattern of action that provides mutual understanding for solving problems or encourages individuals to behave pro-socially when conflicts in self-interest and joint gain arise (Hechter and Opp, 2001). As such, norms are essential for upholding social order. According to Hechter and Opp (2001), social norms refer to standards for behavior in a given situation. Compared with legal rules, social norms are not supported by formal sanctions but are publicly shared. Previous research has found that the perception of social norm-violating behaviors (i.e., the extent to which people can detect social norm-violating behaviors) varies across cultures and societies (Mu et al., 2015). The N400, an event-related potential (ERP) with a negative deflection at approximately 400 ms, is a neural index of the detection of unexpected anomalous stimuli and affective and social incongruent information (Ceballos et al., 2005; Goto et al., 2010). The N400 evoked by a social norm violation task has been found to be more negative in Chinese people than in Americans (Mu et al., 2015). Even at national, regional and state levels, diverse sensitivities to social norm violations are observed (Harrington and Gelfand, 2014).

As the largest developing country, China has witnessed considerable internal labor mobility in the last two decades, accompanied by the implementation of the reform and opening-up policy (Fan, 2003). The traditional Chinese family inherited the intrafamily labor division in which men take the responsibility for working outside the home and women stay at home and raise the offspring. The development of the market economy pushed a great amount of laborers from rural areas into cities and towns to pursue higher pay and better education, which caused an impact on the structure of traditional families and a major transition in the social role of females. According to the investigation of the National Statistics Bureau, the number of female domestic immigrants grew rapidly around 2000, even more rapidly than the growth of male immigrants (National Bureau of Statistics of China, 2005). This great transformation in social roles can cause enormous challenges for female immigrants during the process of social change (Tuccio and Wahba, 2018). A previous study on the gendered division of labor during the transition period in China revealed that the impact of gender stereotypes and underrepresentation in patriarchal society undermined the status of the majority of rural women (Fan, 2003). Female immigrants worldwide seem to be faced with more challenge than male immigrants with regard to their occupational and educational mobility and are reported to have a higher risk of suffering from mental health problems (Fisher and Hood, 1988; Magdol, 2002; Haynie et al., 2006). Women have been found to be more likely to seek help from the others (i.e., professionals or friends) and to be more emotionally interrelated than men when faced with difficulties in life (Ashton and Fuehrer, 1993; Jordan and Revenson, 1999; Addis and Mahalik, 2003; Bosco et al., 2019). As a result, they might be more flexible in adjusting to the changing environment. Nevertheless, limited research has been conducted to investigate how residential mobility impacts the psychological status and social perception of this underrepresented group specifically during the process of social change.

We hypothesized that residential mobility would decrease the detection of social norm violations and that this influence would mainly exist in women due to the demanding adaptation to the change of environment. To test these hypotheses, we conducted three studies with mixed methods, including surveys, behavioral manipulations and neuroscientific approaches (electroencephalography, EEG). In Study 1, we conducted an online survey with a homogenous sample with a predetermined sample size to investigate the association between historical residential mobility and the perception of social norm violations, which was measured by the task developed by Mu et al. (2015). Based on the gendered findings of Study 1, we conducted two separate laboratory EEG experiments to further uncover the neural mechanism of the influence of residential mobility on perceptions of social norm violations (Study 2) and found that the effect was exerted mainly on social norm violations but not on general semantic violations (Study 3).

## Materials and Methods

### Study 1

A total of 175 participants (86 males, 89 females, *M*_age_ = 22.99 years, *SD* = 5.19) were recruited at Sun Yat-sen University. Each participant received 20 yuan in compensation. This sample size allowed us to detect the residential mobility effect with a medium effect size (*r* = 0.30, alpha = 0.05, power = 0.80, number of predictors = 6) and the gender-dependent mobility effect with a medium effect size (*r* = 0.30 vs. 0.10, alpha = 0.05, power = 0.80, allocation ratio = 1) using linear multiple regression (estimated with G^∗^Power software, Faul et al., 2007). Informed consent was obtained from all participants before the experiments were started in all studies. All studies were approved by the ethics committee of the Department of Psychology at Sun Yat-sen University.

The participants were asked to complete an online survey. Subjective socioeconomic status (SES) was measured with McArthur’s Self-Anchoring Scale, which was previously used to link subjective SES with social norm violations (Mu et al., 2015). After reporting background information, the participants were asked to list all the cities or towns to which they had moved and their age when they moved. Cumulative moving times served as the participants’ residential mobility scores. Then, the participants completed a norm violation rating task. The social norm violation rating task contained three types of items (appropriate, weakly social norm violating, and strongly social norm violating) that were adapted from Mu et al. (2015). Each type included eight items, and each item described a behavior in a certain situation. The participants rated the appropriateness of the norm violation items on a scale from 1 (*strongly inappropriate*) to 7 (*strongly appropriate*). In these studies, we report all measures, manipulations and exclusions.

### Study 2

Forty-two female students (*M*_age_ = 19.24 years, *SD* = 1.65, age range: 18–26 years) were recruited from Sun Yat-sen University. All participants were right-handed and had normal or corrected-to-normal vision. Each participant received 50 yuan in compensation.

Prior to the EEG session, the participants completed a survey that included demographic information. The participants were randomly assigned to one of two conditions consistent with the work of Oishi et al. (2013): residential mobility vs. residential stability. The participants in the mobile condition were asked to imagine that they were offered a job that they had always wanted, but it required living in a different city every other year. The participants in the stable condition were asked to imagine obtaining the same dream job, but it required them to remain in the same city for 10 years. Both groups were instructed to write relevant content in 10 min.

Two independent experts read the written responses to the residential mobility/stability manipulation items and rated the concern for relationships with family and friends as well as the overall concern on a 5-point scale (1 = *not at all*, 5 = *extremely*). These two experts also counted words or phrases that were related to loneliness (e.g., isolated, lonely, alone) and decreased social networks. Agreement on the concern scores and word-counting was adequate (*r*s > 0.80). Bonferroni correction was used for multiple comparisons during the *post hoc* comparison.

**FIGURE 1:**
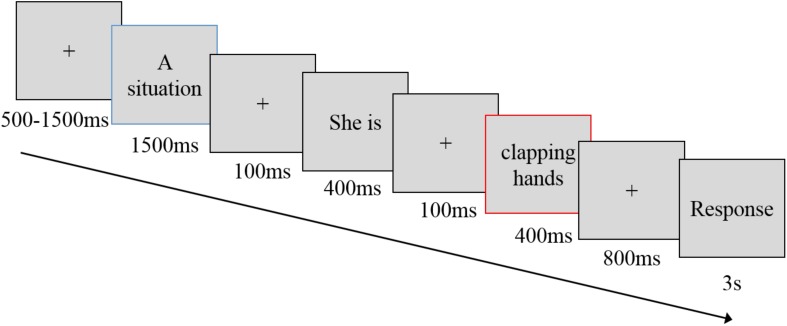
The social norm violation task. Each trial began with a fixation of 500–1500 ms. Then, the first sentence describing a situation was presented (e.g., Elle is at a symphony concert.) for 1500 ms. After a 100-ms fixation, the second sentence depicting a behavior (e.g., she is clapping her hands) was separated into two 400-ms screens with a fixation of 100 ms. After an 800-ms fixation, a response screen was shown for 3 s during which the participants judged the appropriateness of the behavior from 1 (*very inappropriate*) to 4 (*very appropriate*) using their index and middle fingers on both hands on a keyboard, at an 80-cm viewing distance. ERP components were generated in a screen with a red frame.

After the residential mobility manipulation, the participants were asked to imagine the situation that was described in the manipulation and complete a norm violation rating task. In the EEG session, we used a social norm violation task that was based on Mu et al. (2015) (see Figure 1). Thirty-four behaviors (e.g., clapping hands) were presented for three types of situations: appropriate (e.g., at a symphony concert), weakly inappropriate (e.g., in the hotel lobby), and strongly inappropriate (e.g., at a funeral). The participants were asked to judge whether a certain behavior was appropriate in a given situation. For each situation, there were 34 types of behavior. Among the 34 behaviors, 10 were randomly chosen and presented twice. Thus, each situation contained 44 trials. A total of 132 trial (44 behaviors × 3 situations) (Supplementary Tables S7, S8) were randomly assigned across four runs, with each run lasting 4 min. In each run, the participants were instructed to imagine the situation in the opening question and complete the following task. Each trial began with a fixation of 500–1500 ms. Then, the first sentence describing a situation was presented (e.g., Elle is at a symphony concert) for 1500 ms. After a 100-ms fixation, the second sentence that depicted a behavior (e.g., she is clapping her hands) was separated into two 400 ms screens. For example, “she is” was presented for 400 ms followed by a fixation of 100 ms, followed by “clapping hands” presented for 400 ms. After an 800-ms fixation, a response screen was shown for 3 s (at a viewing distance of 80 cm), during which the participants judged the appropriateness of the behavior from 1 (*very inappropriate*) to 4 (*very appropriate*) using the index and middle fingers of both hands on a keyboard. We targeted the ERPs elicited by the screen that presented the behaviors (e.g., clapping hands, screen with a red frame in Figure 1). The entire procedure is depicted in the Supplementary Figure S1. The response buttons were counterbalanced across subjects.

**FIGURE 2:**
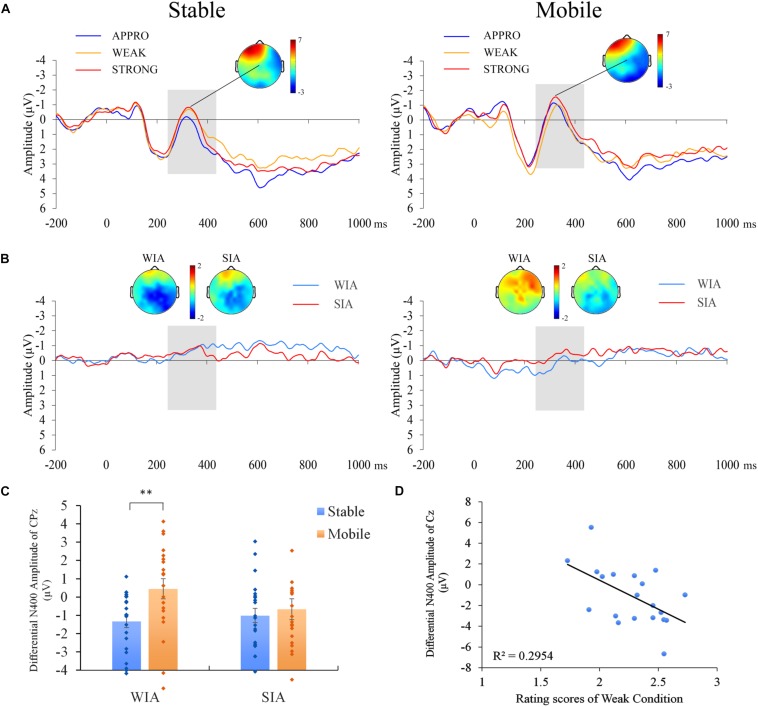
Event-related potential (ERP) results for social norm violations. **(A)** Grand average ERPs for the original appropriate, weak, and strong conditions in the stable residential and mobile residential conditions in the central region. The topography shows the distribution of the N400 effect in the strong condition at 250–450 ms for the stable residential and mobile residential conditions. **(B)** Differential ERPs for the contrast between the weak and appropriate conditions (WIA) and the contrast between the strong and appropriate conditions (SIA) for the stable and mobile conditions. The topographies show the distribution of the differential N400 (WIA and SIA) for the stable and mobile conditions at 250–450 ms. **(C)** Bar chart illustrating the contrast of the N400 effects for WIA and SIA at the central-parietal region (CPz was chosen as a representative electrode) for the 250–450 ms time window. The error bars represent the standard errors. **(D)** Correlation between the differential amplitude to contrast the strong vs. weak conditions in the 250–450 ms time window with subjective rating scores from the weak condition during EEG processing (higher scores correspond to more appropriate behaviors). ^∗∗^*p* < 0.01. Bonferroni corrected for multiple comparisons.

We collected continuous EEG signals using 64 scalp electrodes based on the 10–20 system of the NeuroScan system. The vertical electro-oculogram (VEOG) was recorded from two electrodes located above and below the left eye. The horizontal electro-oculogram (HEOG) was recorded from two electrodes placed 1.5 cm lateral to the left and right external canthi. EEG was amplified (bandpass 0.05–100 Hz) and digitized at a sampling rate of 500 Hz. All data were re-referenced offline to an average mastoid reference and filtered with a 30-Hz low pass. The ERPs in each condition were averaged separately, with an epoch beginning 200 ms prior to the stimulus onset and continuing for 1200 ms. Trials that were contaminated by eye movements and muscle potentials exceeding ±50 μV at HEOG, VEOG, FP1, FPZ and FP2 were excluded from the calculation of the average. The mean of the acceptable trial rate for participants was 82.3% ± 9.3%. The data for each participant were averaged for each situation. The mean amplitude of the N400 component was calculated via electrodes selected from the central-parietal (Cz, C1, C2, CPz, CP1, CP2) regions at the 250- to 450-ms time window (peaking at approximately 350 ms), similar to a previous study in which the N400 was evoked (Fisher et al., 2010; Kutas and Federmeier, 2011) (Figure 2). Voltage topography was used to estimate potential sources of neural responses to violation.

### Study 3

To test whether residential mobility may have an impact on non-social violations, we conducted Study 3 as a control study. Forty female students (*M*_age_ = 19.27 years, *SD* = 1.72, age range: 18–26 years) were recruited from Sun Yat-sen University. All of the participants were right-handed and had normal or corrected-to-normal vision. Each participant received 50 yuan in compensation.

**FIGURE 3:**
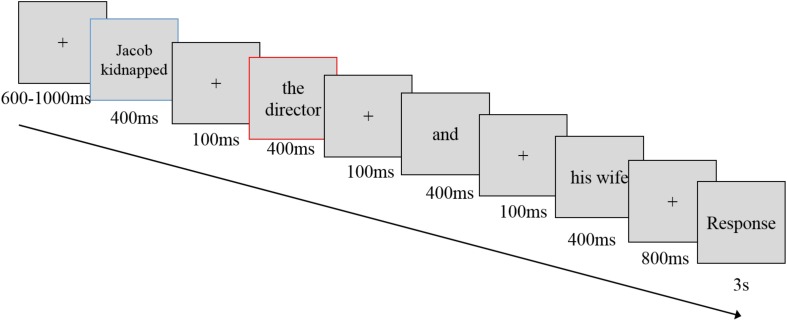
The semantic violation task. Each trial began with a fixation of 550–1000 ms. Then, short phrases segmented from the sentence were shown for 400 ms each, with a 100-ms fixation between two short phrases. After the whole sentence was presented, an 800-ms fixation was shown, followed by a 3-s response screen during which participants judged the correctness of the sentence using their index fingers on both hands on a keyboard, at viewing distance of 80 cm. ERP components were generated in a screen with a red frame.

Prior to the EEG session, the materials and procedures were consistent with those described above for Study 2. The EEG session adopted a semantic violation task based on an established paradigm (see Figure 3). In the semantic violation task, several semantically correct or incorrect sentences were presented, and the participants were asked to judge whether these sentences were right or wrong. Ninety subject-verb-object segmented sentences, including 45 semantically correct and 45 incorrect sentences, were randomly assigned to three runs. Each run lasted 4 min (Supplementary Tables S9, S10). In each run, the participants were instructed to imagine the situation in the opening question and complete the task, which was followed by 30 trials, with 15 trials for each semantic condition. Each trial started with a fixation of 550–1000 ms. Then, short phrases segmented from the sentence were shown for 400 ms each, with a 100-ms fixation between the two short phrases. After the entire sentence was presented, an 800-ms fixation was followed by a 3-s response screen (at a viewing distance of 80 cm), during which the participants judged the correctness of the sentence using the index fingers of both hands on a keyboard. We targeted the ERPs elicited by the screen that presented the objects (e.g., 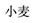
(wheat), screen with a red frame in Figure 3) after the behavior (e.g., 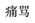 (cursed)). The entire procedure is depicted in the Supplementary Figure S2. The response buttons were counterbalanced across subjects.

EEG data collection and analysis were the same as those described for Study 2, except that trials containing incorrect responses were excluded before filtering, and the data for each participant were averaged for the two semantic conditions (correct vs. incorrect). The mean of the acceptable trials for the participants was 80.7% ± 10.5%.

## Results

### Study 1

We utilized the statistical software SPSS 22.0 to analyze all the data for all the studies. Study 1 obtained the participants’ historical residential mobility data and used a social norm violation rating task to test the relationship between historical residential mobility and perceptions of social norm-violating behavior. The social norm violation rating task was adapted from Mu et al. (2015) and included three types of behavior sorted by the participants’ subjective rating: appropriate, weakly social norm-violating (WSN), and strongly social norm-violating (SSN) behavior. We calculated the scores for WSN and SSN behavior using the original rating scores for these two types of behaviors minus the original appropriate behavior score. The same calculation methods were used consistently in the following studies. Means, standard deviations, and the correlation matrix of all variables are shown in Supplementary Tables S1–S3.

A regression analysis showed that historical residential mobility was significantly associated with the WSN rating scores (WSN: β = 0.17, *p* = 0.029) but marginally significantly associated with the SSN rating scores (SSN: β = 0.15, *p* = 0.051, Table 1). We tested whether gender moderated the relationship between mobility and norm violations, and the results indicated that this relationship was significant for WSN (β = 0.17, *p* = 0.023, Supplementary Table S4). Next, we employed separate regression analysis for the two gender groups (Supplementary Figure S1). For females, higher residential mobility corresponded to a weaker perception of WSN (β = 0.32, *p* = 0.002, Table 2) and SSN (β = 0.25, *p* = 0.020). Residential mobility was not related to norm violations for males (WSN: β = −0.05, *p* = 0.674; SSN: β = 0.05, *p* = 0.691). There was no gender difference in the history of residential mobility (male: 1.40 ± 0.82, female: 1.34 ± 0.85, *t* = 0.46, *p* = 0.645). These results were consistent after controlling for age, location (urban/rural), annual income and subjective SES.

**TABLE 1:** Coefficients from regression analysis in Study 1.

		**WSN**			**SSN**		
		**β**	***t***	***p***	**β**	***t***	***p***
Model 1	Historical mobility	0.17^∗^	2.20	0.029	0.14	1.97	0.051
Model 2	Historical mobility	0.16^∗^	2.09	0.038	0.12	1.52	0.131
	Gender	−0.15^∗^	–2.00	0.047	–0.06	–0.75	0.454
	Age	0.03	0.33	0.744	0.21^∗∗^	2.75	0.007
	Urban-rural	0.02	0.30	0.762	0.05	0.64	0.523
	Income	–0.11	–1.42	0.157	–0.11	–1.42	0.157
	SES	0.09	1.10	0.272	0.12	1.50	0.135

*WSN, the redefined weakly inappropriate condition (original weakly inappropriate - appropriate); SSN, the redefined strongly inappropriate condition (original strongly inappropriate – appropriate). Males were coded as “0,” and females were coded as “1”. Males were set to be the baseline of the analysis. ^∗^*p* < 0.05; ^∗∗^*p* < 0.01.*

**TABLE 2:** Coefficients from regression analyses performed separately for females and males in Study 1.

			**WSN**			**SSN**		
		**Model**	**β**	***t***	***p***	**β**	***t***	***p***
Females	1	Historical mobility	0.32^∗∗^	3.14	0.002	0.25^∗^	2.37	0.020
	2	Historical mobility	0.32^∗∗^	2.95	0.004	0.21	1.93	0.058
		Age	–0.13	–1.16	0.249	0.07	0.64	0.524
		Urban-rural	0.02	0.18	0.860	0.13	1.16	0.249
		Income	0.02	0.21	0.835	0.06	0.53	0.599
		SES	0.02	0.22	0.826	–0.01	–0.08	0.939
Males	3	Historical mobility	–0.05	–0.42	0.674	0.04	0.40	0.691
	4	Historical mobility	–0.04	–0.38	0.708	0.03	0.26	0.794
		Age	0.17	1.50	0.137	0.31^∗∗^	2.88	0.005
		Urban-rural	0.01	0.05	0.962	–0.04	–0.33	0.739
		Income	−0.23^∗^	–2.03	0.046	−0.26^∗^	–2.39	0.019
		SES	0.13	1.17	0.245	0.22^∗^	2.09	0.040

*WSN, the redefined weakly inappropriate condition (original weakly inappropriate – appropriate); SSN, the redefined strongly inappropriate condition (original strongly inappropriate - appropriate). Males were coded as “0,” and females were coded as “1”. Males were set to be the baseline of the analysis. ^∗^*p* < 0.05; ^∗∗^*p* < 0.01.*

We also calculated the weighted residential mobility based on administrative division. We gave different weights to the scores of residential mobility according to the administrative division. Specifically, migrations between cities within provinces were given a weight of 1, and migrations between provinces within the same part of China (i.e., southern/northern China) were given a weight of 2, and migrations between parts of China or between nations were given a weight of 3. Finally, we calculated the total scores of these three types of migrations as the weighted history of residential mobility. With this measure, we found consistent results indicating that a history of residential mobility was significantly correlated with WSN and SSN in females (*r* = 0.319 and 0.247, *p* = 0.002 and 0.02) but not in males (*r* = −0.014 and −0.004, *p* = 0.974 and 0.691, Supplementary Figure S1).

### Study 2

Although the decrease in subjectively appropriate ratings for social norm violations in the residentially mobile context of the behavioral experiment could be explained by a decrease in perceptions of social norm violations, the pattern we found could also result from comparable perception but higher tolerance toward social norm violations. Whether this effect is due to a decrease in detection requires further evidence. Study 2 used the electroencephalography (EEG) technique to investigate the underlying neural mechanisms of residential mobility on social norm violations by examining the N400, the neural marker for detecting social norm violations. If the N400 in the two mobility conditions differs, then the detection of social norm violations is influenced by residential mobility. Because significant interactions were observed between residential mobility and social norm violations for females in Study 1, we focused on females in Study 2. Based on the gendered findings of Study 1, we focused on females in the present study.

For the N400 component elicited by the target screen depicting the behaviors, differential ERPs for the contrasts between the weakly inappropriate (WI)/strongly inappropriate (SI) conditions and the appropriate condition were used to perform the analyses (WIA and SIA, respectively). We performed a 2 × 2 (residential mobility [mobile, stable] × norm [WIA, SIA]) repeated-measures ANOVA for the central and parietal regions. A significant main effect was observed for residential mobility (*F*(1,40) = 4.66, *p* = 0.037, η_p_^2^ = 0.10). The main effect for norms was not significant (*F*(1,40) = 0.69, *p* = 0.412, η_p_^2^ = 0.02; detailed results for each electrode are provided in Supplementary Table S6. A significant interaction was observed between residential mobility and norms in the central and parietal regions (*F*(1,40) = 6.23, *p* = 0.017, η_p_^2^ = 0.14). *Post hoc* analyses revealed that differential ERPs for the WIA were significantly more negative in the stable condition than in the mobile condition (*t*(40) = −3.13, *p* = 0.003), and significant effects were not observed between the two residential mobility conditions for the differential ERPs for the SIA (*t*(40) = −0.55, *p* = 0.583), which indicated that residential mobility may decrease perceptions of weak violations (see Figure 2).

To explore the relationship between the participants’ subjective ratings (Supplementary Table S5) and neural responses to the WI condition, we performed a correlation analysis. The results showed that the differential amplitudes between the SI and WI conditions (SIWI) were negatively related to the subjective ratings for weak violations in the mobile condition (*r* = −0.56, *p* = 0.011). This association was not detected for the residentially stable condition (*r* = 0.27, *p* = 0.226). Hierarchical regression analyses further confirmed that residential mobility moderated the relation between the weak violation rating scores and the differential ERPs for SIWI in the central and parietal regions (β = −0.403, *p* = 0.004).

### Study 3

To test whether residential mobility may have an impact on non-social violations, we conducted Study 3 in which a semantic violation task (Mu et al., 2015) was used.

**FIGURE 4:**
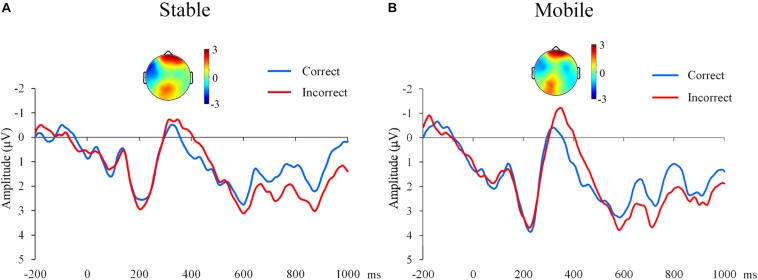
Event-related potential (ERP) results for semantic violations. **(A)** Grand average ERPs for the semantically correct and incorrect conditions for the stable residential condition in the central region. The topography shows the distribution of the N400 effect for the incorrect condition at 250–450 ms in the stable residential condition. **(B)** Grand average ERPs for the semantically correct and incorrect conditions in the mobile residential condition in the central region. The topography shows the distribution of the N400 effect for the incorrect condition at 250–450 ms in the mobile residential condition.

In this study, participants were randomly assigned to either the residential mobility or stability conditions and completed a sematic violation task. The accuracy rates for all participants for the semantic violation task were higher than 80%. For the N400 amplitude, we employed a 2 × 2 (residential mobility [mobile, stable] × semantic violation [correct, incorrect]) repeated-measures ANOVA. A more negative N400 was elicited in the semantically incorrect condition than in the correct condition (*F*(1,38) = 9.73, *p* = 0.003, η_p_^2^ = 0.20, see Figure 4), and a main effect was not observed for residential mobility and the interaction between residential mobility and violation (residential mobility: *F*(1,38) = 0.40, *p* = 0.529, η_p_^2^ = 0.01; interaction: *F*(1,38) = 1.96, *p* = 0.170, η_p_^2^ = 0.05).

## Discussion

Our research examined the relationship between historical residential mobility and social norm violations. In addition, we manipulated residential mobility and found that it influenced neural responses to social norm violations.

### Residential Mobility and Social Norm Violations in Females

Our results showed that historical residential mobility was correlated with decreased detection of both weak and strong social norm-violating behaviors among females in Study 1. After priming with residential mobility, the neural component of social norm violation was subdued in the context in which the violation was weak. The findings of the present study suggest that the perception of social norms is flexible in females, which might contribute to their coping with a changing environment. Previous studies on gendered vulnerabilities to climate change revealed that females are most vulnerable among vulnerable groups to extreme climatic situations due to their underrepresentation in the distribution of social resources (Ferdous and Mallick, 2018; Goodrich et al., 2019). Similar situations occur during the course of mobility, especially in the context of traditional Chinese culture, such as the traditional gendered norms of women staying at home and inequity in obtaining opportunities for education (Fan, 2003). Additionally, previous research has found that underrepresented minority groups are more other-oriented, and at public scientific institutions, research microcultures act as socialization contexts for underrepresented science students and are integrated into their personal science norms (Thoman et al., 2017). In summary, due to their inadequate power to exert a strong influence on the surrounding environment, women might adopt new social norms that are formulated within a more diverse group, and this impact is apparent at the stage of detection. However, insufficient research has been conducted to investigate how the psychological and social status of female teenagers changes during the process of residential mobility, which is a worthwhile topic for future studies.

Nevertheless, we did not find significant associations between residential mobility and perceptions of strong social norm-violating behavior among females in Study 2. This may be because the effect for strong violations was not as strong as that for weak violations and may not have been detected in the current sample size with 80% power. We assumed that strong social norm violations have some overlap with moral norm violations, whereas weak social norm violations do not. Moral norms are internalized, and regardless of the circumstances, behavior that violates moral norms is wrong (Bicchieri and Muldoon, 2014). As such, perceptions of moral norm violations should be inflexible. With regard to social norms, people can establish an association between a situation and a normative behavior, and these associations can strengthen such that normative behavior occurs automatically when a situation is depicted (Aarts and Dijksterhuis, 2003). In such circumstances, if a behavior transgresses a norm, the behavior is a strong violation that can be easily detected. On the other hand, moral norm violations hurt others physically (Royzman et al., 2009) as well as emotionally (Bicchieri, 2005). Some SSN behaviors, such as “laughing out loud at a funeral,” can emotionally harm the offended. Thus, perceptions of strong social norm violations may be less dependent on social context than weak social norm violations. The effect of residential mobility on perceptions of strong social norm-violating behaviors requires further examination.

### Neural Mechanism Underlying the Residential Mobility Effect on Social Norm Violations

At the neural level, WI behavior evoked more negative differential N400s in the stable residential condition than in the mobile residential condition in females, whereas no differences for strong social norm violations were observed in either residential mobility condition. In previous research, N400 effects were observed for social inconsistencies, such as stereotypes (White et al., 2009) and affective incongruities (Goto et al., 2013; Fong et al., 2014), as well as non-social incongruities, including semantic violations (Proverbio and Riva, 2009) and the Stroop test (Rebai et al., 1997). In our study, the N400 results may have reflected the detection of social norm violations, as indicated by the inconsistencies between an expected and an observed behavior in a given situation. Furthermore, the negative correlation between the differential ERPs for the SI and WI conditions and the subjective rating of weak violations in the mobile condition indicate that mobility weakened the detection of weak social norm violations. These results sufficiently suggest that the detection of social norm violations is affected by mobility. If mobility only affected people’s tolerance, they could still detect inappropriate behaviors; thus, top-down regulation would lead them to make appropriate ratings. However, according to our findings, the detection of weakly violating behaviors was modified in the mobile residential condition. Thus, the reason why mobility increases the subjective ratings of weak violations at the behavioral level is that mobility reduces people’s perception of weak violation situations rather than that mobility increases their tolerance to weak violation situations.

Our ERP findings from Study 2 and our behavioral data from Study 1 consistently suggest that residential mobility decreases the detection of weak social norm violations. It should also be noted that residential mobility influences external behaviors as well as psychological processes at the neurobiological level. Previous research has shown that diversified experiences lead to cognitive flexibility and norm violations (Ritter et al., 2012). Individuals who are in residentially mobile contexts may become involved in diverse contexts and may be exposed to unusual issues or situations. As a result, they may become more cognitively flexible with social norm violations, meaning that they may become less sensitive to these violations. Notably, residential mobility did not influence the detection of semantic violations, as shown in Study 3, indicating that the impact of residential mobility on the detection of social norm violations follows a specific pattern.

### Theoretical Implications and Future Directions

Social changes have an impact on individuals through mixed approaches, and individuals respond differently based on both the micro and macro culture they encounter. The findings of the present study suggest that studying the effect of residential mobility on social cognition would allow gender differences to be taken into account. Changes in social cognition and social status might be passed to the next generation by interaction among family members (McGinn et al., 2018; Tuccio and Wahba, 2018). Females play a significant role in influencing the values and perspectives of children due to their responsibility to teach and raise the next generations, especially in the context of traditional Chinese culture (King and Bond, 1985). More studies could be conducted to investigate how the impact of residential mobility on females’ social cognition is transmitted and passed on intergenerationally.

Psychological consequences may lead to behavioral changes. Although the reduced perception of social norm violations might help women adapt to changing environments and blend in, it may also lead to a “side effect,” such as an increase in counter-normative behaviors. Studies have shown that low moral sensitivity is related to increased antisocial behavior (Blair, 1997; Thornberg and Jungert, 2013). At the neural level, the dorsal and ventral prefrontal cortex, the amygdala and the angular gyrus are activated during moral judgment tasks and represent some of the primary areas in which antisocial individuals are functionally or structurally impaired, suggesting that behaving antisocially is partially caused by impairments in the moral judgment-related regions of the brain (Raine and Yang, 2006). In other words, failing to perceive a behavior as a moral violation because of brain impairment may lead to antisocial behavior. Our studies found that residential mobility influences the perception of social norm violations at the psychological level. Future research should examine whether decreased sensitivity to social norms due to mobility causes social norm-violating behaviors.

Importantly, the psyche and social ecology are mutual, and this mutual constitution is the nexus of socioecological psychology (Oishi and Graham, 2010). Our research found that residential mobility decreased an individual’s detection of social norm violations, which may lead to social norm-violating behavior. Social norm violators in a residentially stable context may encounter harsh punishments and may not be able to maintain their face, which may encourage more frequent moves. In this case, social norm violations may contribute to higher residential mobility in a society.

Another issue that warrants attention is the dynamic influence of residential mobility on individual judgments of norm violations, specifically in societies that have been developing at a rapid pace (e.g., China). The frequency of residential mobility coded in the present study takes into account the mobile range (i.e., moving within provinces or moving across provinces), and the results remain consistent. It is worth investigating in the future whether mobility of a broader range (e.g., international or cross-cultural mobility) would influence the perception of social norm violation differently. During the globalization era, the consequences of residential mobility warrant further examination. This area of research may assist in resolving certain questions in human society.

## Data Availability Statement

The datasets underlying the findings described and used to reach the conclusions of the manuscript are included in the Supplementary Material. Other data in this project is also available on request to the corresponding author for qualified researchers.

## Ethics Statement

All participants gave written informed consent, and the study was approved by the Department of Psychology of Sun Yat-sen University Ethics Committee.

## Author Contributions

QK and SL designed the research. QK, YZ, and YX conducted the experiments. QK, ZK, and SL analyzed the data. QK, ZK, MY, LH, and SL wrote the manuscript. All authors commented on the manuscript.

## Conflict of Interest

The authors declare that the research was conducted in the absence of any commercial or financial relationships that could be construed as a potential conflict of interest.
