# Supplementary material for: Residential Mobility Decreases Neural Responses to Social Norm Violation
Source: Front Psychol. 2019 Nov 28;10:2654. doi: 10.3389/fpsyg.2019.02654 (PMC6894357; doi:10.3389/fpsyg.2019.02654)
Supplement: Supplementary file 1 [file Data_Sheet_1.docx]

**Supplementary Information**

**Residential Mobility Decreases Detections of Social Norm Violation**

**Siyang Luo^1,*^,** **Qianting Kong^1,*^,** **Zijun Ke^1,*^, Yiyi Zhu^1^,** **Liqin Huang^1^, Meihua Yu^1^, Ying Xu^1^**

**^1^Department of Psychology,**

**Guangdong Key** **Laboratory of Social Cognitive Neuroscience and Mental Health,**

**Guangdong Provincial Key Laboratory of Brain Function and Disease,**

**Sun Yat-Sen University**

**Guangzhou 510006, China**

**Supplementary Information:**

**Supplementary methods & results**

**Tables S1-10**

**Figures S1-2**

Table S1. Descriptive and correlation matrix results in study 1.

|  | Mean±SD | Historical mobility | Gender | Age | Urban-rural | Income | SES | WSN | SSN |
| --- | --- | --- | --- | --- | --- | --- | --- | --- | --- |
| Historical mobility | 1.37±0.83 | 1 | -0.035 | 0.035 | .170* | 0.037 | 0.066 | .167* | 0.123 |
| Gender | - | -0.035 | 1 | -0.028 | -0.142 | 0.147 | 0.022 | -.167* | -0.08 |
| Age | 22.99±5.19 | 0.035 | -0.028 | 1 | 0.067 | .253** | -0.063 | 0.014 | .194** |
| Urban-rural | 3.35±1.69 | .170* | -0.142 | 0.067 | 1 | -.300** | -.260** | -0.003 | 0.077 |
| Income | 3.37±1.60 | 0.037 | 0.147 | .253** | -.300** | 1 | .174* | -0.104 | -0.045 |
| SES | 5.47±1.42 | 0.066 | 0.022 | -0.063 | -.260** | .174* | 1 | 0.074 | 0.086 |
| WSN | -2.90±0.87 | .167* | -.167* | 0.014 | -0.003 | -0.104 | 0.074 | 1 | .582** |
| SSN | -4.19±1.02 | 0.123 | -0.08 | .194** | 0.077 | -0.045 | 0.086 | .582** | 1 |

WSN (original weakly inappropriate - appropriate); SSN (original strongly inappropriate - appropriate).

Table S2. Descriptive and correlation matrix results in female group in study 1.

|  | Mean±SD | Historical mobility | Age | Urban-rural | Income | SES | WSN | SSN |
| --- | --- | --- | --- | --- | --- | --- | --- | --- |
| Historical mobility | 1.34±0.85 | 1 | -0.027 | 0.208 | -0.06 | 0.072 | .313** | .253* |
| Age | 22.85±4.68 | -0.027 | 1 | 0.036 | .349** | -0.105 | -0.106 | 0.11 |
| Urban-rural | 3.11±1.52 | 0.208 | 0.036 | 1 | -.346** | -.321** | -0.044 | 0.132 |
| Income | 3.60±1.75 | -0.06 | .349** | -.346** | 1 | 0.167 | -0.009 | 0.072 |
| SES | 5.51±1.45 | 0.072 | -0.105 | -.321** | 0.167 | 1 | 0.08 | 0 |
| WSN | -3.05±0.90 | .313** | -0.106 | -0.044 | -0.009 | 0.08 | 1 | .481** |
| SSN | -4.27±0.95 | .253* | 0.11 | 0.132 | 0.072 | 0 | .481** | 1 |

WSN (original weakly inappropriate - appropriate); SSN (original strongly inappropriate - appropriate).

Table S3. Descriptive and correlation matrix results in male group in study 1.

|  | Mean±SD | Historical mobility | Age | Urban-rural | Income | SES | WSN | SSN |
| --- | --- | --- | --- | --- | --- | --- | --- | --- |
| Historical mobility | 1.40±0.82 | 1 | 0.089 | 0.132 | 0.182 | 0.061 | -0.014 | -0.004 |
| Age | 23.14±5.69 | 0.089 | 1 | 0.083 | 0.177 | -0.027 | 0.117 | .255* |
| Urban-rural | 3.59±1.84 | 0.132 | 0.083 | 1 | -.232* | -0.208 | -0.013 | 0.017 |
| Income | 3.13±1.40 | 0.182 | 0.177 | -.232* | 1 | 0.181 | -0.186 | -0.156 |
| SES | 5.44±1.40 | 0.061 | -0.027 | -0.208 | 0.181 | 1 | 0.079 | 0.171 |
| WSN | -2.76±0.82 | -0.014 | 0.117 | -0.013 | -0.186 | 0.079 | 1 | .683** |
| SSN | -4.10±1.09 | -0.004 | .255* | 0.017 | -0.156 | 0.171 | .683** | 1 |

WSN (original weakly inappropriate - appropriate); SSN (original strongly inappropriate - appropriate).

Table S4. Coefficients from regression analysis of the gender moderation effect on WSN in study 1.

|  | β | t | p | β | t | p |
| --- | --- | --- | --- | --- | --- | --- |
| DV: WSN |  |  |  |  |  |  |
| Residential mobility | 0.13 | 1.68 | 0.095 | 0.12 | 1.56 | 0.121 |
| Gender | -0.17* | -2.31 | 0.022 | -0.15* | -2.04 | 0.043 |
| Residential mobility × Gender | 0.17* | 2.29 | 0.023 | 0.17* | 2.18 | 0.031 |
| Age |  |  |  | 0.04 | 0.46 | 0.648 |
| Urban-rural |  |  |  | 0.02 | 0.24 | 0.814 |
| Income |  |  |  | -0.11 | -1.33 | 0.187 |
| SES |  |  |  | 0.08 | 1.07 | 0.287 |

*Note.* Male was coded as “0” and female was coded as “1”. Male was set to be the baseline of the analysis.

^*^ p < 0.05; ^**^ p < 0.01; ^***^ p < 0.001.

Table S5. Rating scores on the social norm violation task (mean ± SD).

|  | Residential Stable | Residential Mobile | Total |
| --- | --- | --- | --- |
| WSN | -1.36 ± 0.28 | -1.26 ± 0.41 | -1.31 ± 0.35 |
| SSN | -1.92 ± 0.32 | -1.91 ± 0.47 | -1.92 ± 0.39 |

*Note.* WSN = the redefined weakly inappropriate condition (original weakly inappropriate - appropriate); SSN = the redefined strongly inappropriate condition (original strongly inappropriate - appropriate).

Table S6. N400 amplitude in each electrode during the social norm violation task (mean ± SD).

|  | Low Residential Mobility | | | | | | High Residential Mobility | | | | | |
| --- | --- | --- | --- | --- | --- | --- | --- | --- | --- | --- | --- | --- |
|  | Appropriate | | WSN | | SSN | | Appropriate | | WSN | | SSN | |
|  | Mean | SD | Mean | SD | Mean | SD | Mean | SD | Mean | SD | Mean | SD |
| FP1 | 5.04 | 3.49 | 5.51 | 3.29 | 5.79 | 2.46 | 5.34 | 2.83 | 5.59 | 3.22 | 5.29 | 4.28 |
| FPZ | 4.92 | 3.52 | 5.24 | 3.46 | 5.46 | 2.96 | 4.57 | 3.08 | 5.07 | 3.48 | 4.57 | 4.71 |
| FP2 | 3.39 | 3.92 | 3.57 | 3.81 | 3.68 | 3.50 | 3.19 | 3.12 | 4.06 | 3.41 | 3.35 | 4.57 |
| AF3 | 4.90 | 3.23 | 4.93 | 3.15 | 5.40 | 2.53 | 4.43 | 2.87 | 5.11 | 3.45 | 4.53 | 4.11 |
| AF4 | 3.08 | 3.98 | 3.14 | 3.69 | 3.28 | 3.32 | 2.38 | 3.43 | 3.34 | 3.76 | 2.50 | 4.74 |
| AF7 | 5.83 | 2.71 | 6.03 | 2.69 | 6.28 | 2.03 | 5.45 | 2.92 | 6.01 | 3.39 | 5.88 | 3.83 |
| AF8 | 0.61 | 4.30 | 0.82 | 3.83 | 0.86 | 3.91 | 0.73 | 2.55 | 1.76 | 3.37 | 1.04 | 4.02 |
| F7 | 5.09 | 2.67 | 4.76 | 2.30 | 4.95 | 1.93 | 4.62 | 2.88 | 5.25 | 3.22 | 4.97 | 3.68 |
| F5 | 4.44 | 2.50 | 4.21 | 2.72 | 4.69 | 2.11 | 3.90 | 2.74 | 4.68 | 3.21 | 4.05 | 3.67 |
| F3 | 4.25 | 2.79 | 4.21 | 2.77 | 4.25 | 2.03 | 3.61 | 2.92 | 4.44 | 3.40 | 3.78 | 3.83 |
| F1 | 3.91 | 3.66 | 3.70 | 3.80 | 3.99 | 2.80 | 3.02 | 3.20 | 3.93 | 3.46 | 3.23 | 4.12 |
| FZ | 3.87 | 4.32 | 3.38 | 4.27 | 4.00 | 3.49 | 2.79 | 3.39 | 3.31 | 4.06 | 2.59 | 4.71 |
| F2 | 3.38 | 4.20 | 2.58 | 4.28 | 3.02 | 3.46 | 1.96 | 3.58 | 2.73 | 3.96 | 1.72 | 4.81 |
| F4 | 2.03 | 4.09 | 1.58 | 3.83 | 1.83 | 3.53 | 1.13 | 3.73 | 1.83 | 3.79 | 1.15 | 4.49 |
| F6 | 1.08 | 4.09 | 0.86 | 3.91 | 0.79 | 3.47 | 0.51 | 3.04 | 1.58 | 3.30 | 0.61 | 3.84 |
| F8 | 0.13 | 3.72 | -0.07 | 3.48 | -0.24 | 3.58 | 0.24 | 2.50 | 1.29 | 2.91 | 0.07 | 3.43 |
| FT7 | 3.55 | 2.13 | 3.15 | 2.20 | 3.25 | 1.63 | 3.25 | 2.70 | 3.79 | 3.05 | 3.31 | 3.03 |
| FC5 | 3.48 | 2.32 | 2.73 | 2.43 | 3.30 | 1.87 | 2.85 | 2.56 | 3.40 | 2.97 | 2.98 | 3.31 |
| FC3 | 3.48 | 3.29 | 2.59 | 3.16 | 3.18 | 2.34 | 2.54 | 2.70 | 3.13 | 3.21 | 2.47 | 3.93 |
| FC1 | 3.28 | 3.82 | 2.44 | 3.87 | 3.14 | 3.29 | 2.03 | 2.83 | 2.52 | 3.37 | 1.60 | 4.11 |
| FCZ | 2.85 | 4.33 | 2.10 | 4.71 | 2.77 | 3.75 | 1.47 | 3.05 | 2.16 | 3.91 | 1.40 | 4.60 |
| FC2 | 2.49 | 4.47 | 1.32 | 4.32 | 1.75 | 3.67 | 0.78 | 3.18 | 1.43 | 3.73 | 0.42 | 4.69 |
| FC4 | 1.75 | 4.48 | 0.69 | 4.01 | 0.69 | 3.77 | 0.35 | 3.30 | 1.00 | 3.19 | -0.11 | 4.58 |
| FC6 | 1.10 | 4.11 | 0.29 | 3.68 | 0.16 | 3.45 | -0.30 | 2.68 | 0.94 | 3.22 | -0.27 | 3.93 |
| FT8 | 0.08 | 3.41 | -0.53 | 2.92 | -0.52 | 3.38 | -0.18 | 2.28 | 0.73 | 2.66 | -0.65 | 3.26 |
| T7 | 2.39 | 2.16 | 1.92 | 2.10 | 2.09 | 1.88 | 2.00 | 2.09 | 2.36 | 2.34 | 1.94 | 2.42 |
| C5 | 2.83 | 2.68 | 2.11 | 2.57 | 2.37 | 2.06 | 2.09 | 2.32 | 2.43 | 2.75 | 1.87 | 2.98 |
| C3 | 3.00 | 3.47 | 1.82 | 3.22 | 2.32 | 2.83 | 1.74 | 2.53 | 2.15 | 3.11 | 1.42 | 3.67 |
| C1 | 2.66 | 3.98 | 1.46 | 3.88 | 2.04 | 3.35 | 0.96 | 2.82 | 1.86 | 3.22 | 0.69 | 4.01 |
| CZ | 2.29 | 4.20 | 0.96 | 4.52 | 1.28 | 3.93 | 0.73 | 3.08 | 1.18 | 3.46 | 0.07 | 4.29 |
| C2 | 2.08 | 4.55 | 0.62 | 4.26 | 1.34 | 3.82 | 0.26 | 3.15 | 0.98 | 3.33 | -0.03 | 4.34 |
| C4 | 1.46 | 4.53 | 0.31 | 4.48 | 0.48 | 3.46 | 0.00 | 3.02 | 0.53 | 2.98 | -0.74 | 4.09 |
| C6 | 0.92 | 4.21 | -0.02 | 3.59 | 0.15 | 3.57 | -0.16 | 2.72 | 0.36 | 2.89 | -0.75 | 3.53 |
| T8 | 0.33 | 3.64 | -0.67 | 3.04 | -0.41 | 3.06 | -0.46 | 2.23 | 0.35 | 2.40 | -0.77 | 2.97 |
| TP7 | 1.71 | 2.28 | 1.32 | 2.22 | 1.60 | 2.29 | 1.23 | 1.80 | 1.40 | 2.04 | 1.14 | 1.80 |
| CP5 | 2.75 | 2.65 | 1.76 | 2.67 | 2.04 | 2.70 | 1.77 | 2.11 | 1.82 | 2.60 | 1.23 | 2.87 |
| CP3 | 2.93 | 3.58 | 1.67 | 3.46 | 2.14 | 3.02 | 1.89 | 2.61 | 1.96 | 3.13 | 0.93 | 3.57 |
| CP1 | 2.95 | 4.01 | 1.35 | 3.86 | 1.90 | 3.52 | 1.44 | 2.73 | 1.83 | 3.24 | 0.67 | 4.14 |
| CPZ | 2.50 | 4.32 | 1.14 | 4.02 | 1.34 | 3.86 | 0.82 | 2.96 | 1.51 | 3.17 | 0.31 | 4.22 |
| CP2 | 2.20 | 4.24 | 0.59 | 3.98 | 1.55 | 3.89 | 0.69 | 2.96 | 1.10 | 3.19 | 0.11 | 4.23 |
| CP4 | 2.23 | 4.44 | 0.65 | 3.97 | 1.15 | 3.72 | 0.56 | 2.79 | 0.91 | 2.78 | -0.27 | 3.78 |
| CP6 | 1.60 | 4.08 | 0.15 | 3.35 | 0.65 | 3.30 | 0.09 | 2.49 | 0.42 | 2.47 | -0.83 | 3.40 |
| TP8 | 0.90 | 2.89 | -0.32 | 2.87 | 0.25 | 2.98 | -0.35 | 1.85 | -0.20 | 1.55 | -0.88 | 2.27 |
| P7 | 1.50 | 2.18 | 0.67 | 2.57 | 0.97 | 2.65 | 0.39 | 1.77 | 0.58 | 1.99 | 0.01 | 1.38 |
| P5 | 2.50 | 2.95 | 1.81 | 3.26 | 2.15 | 3.56 | 1.58 | 2.12 | 1.53 | 2.38 | 0.90 | 2.35 |
| P3 | 3.18 | 3.55 | 1.97 | 3.27 | 2.40 | 3.30 | 1.97 | 2.31 | 2.04 | 2.88 | 1.30 | 3.22 |
| P1 | 3.31 | 3.82 | 1.90 | 3.54 | 2.12 | 3.60 | 1.65 | 2.67 | 2.12 | 3.03 | 1.18 | 3.70 |
| PZ | 2.95 | 4.01 | 1.47 | 3.87 | 2.00 | 3.79 | 1.03 | 2.66 | 1.83 | 2.90 | 0.79 | 3.98 |
| P2 | 2.96 | 4.21 | 1.49 | 3.77 | 1.84 | 3.82 | 0.77 | 2.74 | 1.48 | 2.79 | 0.50 | 3.93 |
| P4 | 2.94 | 3.98 | 1.76 | 3.54 | 1.94 | 3.62 | 0.82 | 2.52 | 0.93 | 2.58 | 0.03 | 3.45 |
| P6 | 2.80 | 3.67 | 1.28 | 3.21 | 1.78 | 3.31 | 0.21 | 2.09 | 0.40 | 2.25 | -0.61 | 2.81 |
| P8 | 1.64 | 3.13 | 0.33 | 2.79 | 0.76 | 3.00 | -0.61 | 1.88 | -0.58 | 2.03 | -1.43 | 2.43 |
| PO7 | 1.08 | 2.30 | 0.40 | 2.69 | 0.58 | 2.86 | 0.47 | 2.08 | 0.41 | 2.44 | -0.35 | 2.39 |
| PO5 | -0.95 | 1.80 | -1.09 | 1.79 | -0.98 | 1.99 | -0.98 | 1.56 | -0.94 | 1.69 | -1.65 | 1.42 |
| PO3 | 3.01 | 3.45 | 2.06 | 3.43 | 2.02 | 3.74 | 1.80 | 2.45 | 1.90 | 2.91 | 1.18 | 3.01 |
| POZ | 2.77 | 3.97 | 1.70 | 3.52 | 1.79 | 3.75 | 0.71 | 2.30 | 1.00 | 2.91 | 0.28 | 3.24 |
| PO4 | 2.98 | 3.92 | 1.57 | 3.22 | 1.98 | 3.69 | 0.33 | 2.27 | 0.76 | 2.52 | -0.17 | 3.19 |
| PO6 | -0.12 | 1.74 | -1.21 | 1.94 | -1.02 | 2.13 | -1.68 | 1.75 | -1.63 | 1.73 | -2.42 | 2.20 |
| PO8 | 2.17 | 3.50 | 0.62 | 3.43 | 1.24 | 3.43 | -0.63 | 2.16 | -0.75 | 2.54 | -1.50 | 2.77 |
| O1 | 1.31 | 2.85 | 0.76 | 2.98 | 0.68 | 2.97 | 0.21 | 2.40 | 0.30 | 2.90 | -0.41 | 2.44 |
| OZ | 0.94 | 2.79 | 0.03 | 2.60 | 0.06 | 2.87 | -0.48 | 1.99 | -0.48 | 2.56 | -1.17 | 2.31 |
| O2 | 1.18 | 2.79 | 0.15 | 2.51 | 0.51 | 2.94 | -0.79 | 1.95 | -0.79 | 2.40 | -1.55 | 2.34 |

*Note.* The N400 amplitude of the three conditions (appropriate, weak, and strong) in the social norm violation task was calculated at the 250–450 ms time window.

Table S7. Items in the Social Norm Violation Rating Task in English.

| Appropriate | Weakly Social Norm-Violating | Strongly Social Norm-Violating |
| --- | --- | --- |
| Zhang Lei is cycling in the bike lane. | Zhang Lei is cycling on the highway. | Zhang Lei is cycling at the pedestrians. |
| Zhang Yan is dancing at a Tango lesson. | Zhang Yan is dancing in the art museum. | Zhang Yan is dancing at the subway platform. |
| Li Jie is flirting in the bar with friends. | Li Jie is flirting in the doctor’s office. | Li Jie is flirting at the post office. |
| Wang qiang is joking in the bar with friends. | Wang qiang is joking with the boss in the meeting room. | Wang qiang is joking with strangers in the airplane. |
| Wang Xiulan is kissing at a wedding. | Wang Xiulan is kissing in the doctor’s office. | Wang Xiulan is kissing on the bus. |
| Li Yan is taking photos at a wedding. | Li Yan is taking photos in the funeral. | Li Yan is taking photos at the class. |
| Wang qiang is praying at church. | Wang qiang is praying in the choir performance. | Wang qiang is praying in the park. |
| Wang Juan is singing in a choir performance. | Wang Juan is singing at the hospital. | Wang Juan is singing on the pedestrians. |
| Zhang Tao is stretching arms at the gym. | Zhang Tao is stretching arms when listening to the lecture. | Zhang Tao is stretching arms in the restaurant. |
| Li Li is bargaining at a real estate agent office. | Li Li is bargaining in the church. | Li Li is bargaining in the taxi. |
| Liu Fang is breastfeeding at home. | Liu Fang is breastfeeding in the library. | Liu Fang is breastfeeding in the park. |
| Wang Yong is eating pancakes in the school canteen. | Wang Yong is eating pancakes at the class. | Wang Yong is eating pancakes on the bus. |
| Wang Tao is arguing at the philosophy seminar. | Wang Tao is arguing in the elevator. | Wang Tao is arguing in the park. |
| Li Ming is hugging with his girlfriend in his living room. | Li Ming is hugging with his girlfriend in the meeting room. | Li Ming is hugging with his girlfriend at the bank. |
| Zhang Jie is laughing out loud in the bar. | Zhang Jie is laughing out loud in the funeral. | Zhang Jie is laughing out loud on the bus. |
| Zhang Lei is taking on cell phone at home | Zhang Lei is taking on cell phone at the theater. | Zhang Lei is taking on cell phone at the post office. |
| Li Xia is crying loud in the funeral. | Li Xia is crying loud in the class. | Li Xia is crying loud on the bus. |
| Wang Jun is cursing in his room. | Wang Jun is cursing at the family gathering. | Wang Jun is cursing in the park. |
| Wang Juan is holding hands at the theater. | Wang Juan is holding hands with her boyfriend at the business meeting. | Wang Juan is holding hands with her boyfriend at the bank. |
| Wang Xiulan is sleeping on the sofa at home. | Wang Xiulan is sleeping at the class. | Wang Xiulan is sleeping on the bench in the park. |
| Li Jing is texting in her living room. | Li Jing is texting in the church. | Li Jing is texting in the gym. |
| Wang Fang is chatting at a coffee bar. | Wang Fang is chatting at the mediation class. | Wang Fang is chatting at the theater. |
| Zhang Wei is yelling at the rock concert. | Zhang Wei is yelling in the library. | Zhang Wei is yelling on the metro. |
| Wang Xiuying is sunbathing on the beach. | Wang Xiuying is sunbathing in the funeral. | Wang Xiuying is sunbathing in the campus. |
| Li Wei is yawning at his bedroom. | Li Wei is yawning with the boss in the meeting room. | Li Wei is yawning when listening to the lecture. |
| Li Na is putting on lipstick at her bathroom. | Li Na is putting on lipstick in the church. | Li Na is putting on lipstick at the bank. |
| Li Xia is changing her baby’s diapers at home. | Li Xia is changing her baby’s diapers at the hotel lobby. | Li Xia is changing her baby’s diapers in the park. |
| Li Qiang is playing guitar in the band performance. | Li Qiang is playing guitar in the hospital. | Li Qiang is playing guitar on the metro platform. |
| Liu Wei is chewing gum in his living room. | Liu Wei is chewing gum when attending the business negotiation. | Liu Wei is chewing gum when attending the awards ceremony. |
| Wang Lei is listening to MP3 on the train. | Wang Lei is listening to MP3 in the class. | Wang Lei is listening to MP3 at the bar. |
| Zhang Li is brushing teeth in her bathroom. | Zhang Li is brushing teeth in the hotel. | Zhang Li is brushing teeth in the public lavatory. |
| Li Fang is clapping hands in a choir performance. | Li Fang is clapping hands in the funeral. | Li Fang is clapping hands in the hotel lobby. |
| Wang Fang is painting at the art studio. | Wang Fang is painting in the hospital. | Wang Fang is painting at the street corner. |
| Li Mei is reading books in the library. | Li Mei is reading books in the church. | Li Mei is reading books in the supermarket. |

*Note.* A Chinese version was used in our study.

Table S8. Items in the Social Norm Violation Rating Task in Chinese.

| Appropriate | Weakly Social Norm-Violating | Strongly Social Norm-Violating |
| --- | --- | --- |
| 张磊在自行车道上骑车 | 张磊在高速公路上骑车 | 张磊在城市人行道上骑车 |
| 张艳在探戈课上跳舞 | 张艳在美术馆里跳舞 | 张艳在地铁月台上跳舞 |
| 李杰在酒吧调情 | 李杰在医生办公室调情 | 李杰在邮局调情 |
| 王强和朋友正在酒吧讲笑话 | 王强和老板在会议室讲笑话 | 王强和陌生人在飞机上讲笑话 |
| 王秀兰在朋友婚礼上接吻 | 王秀兰在医生办公室接吻 | 王秀兰在公交车上接吻 |
| 李艳在朋友婚礼上照相 | 李艳在葬礼上照相 | 李艳在课堂上照相 |
| 王强在教堂祈祷 | 王强在交响乐会上祈祷 | 王强在公园祈祷 |
| 王娟在参加合唱团演出唱歌 | 王娟在医院唱歌 | 王娟在人行道上唱歌 |
| 张涛在体育馆伸展手臂 | 张涛在听课堂讲座伸展手臂 | 张涛在饭店里伸展手臂 |
| 李丽在房产经纪人办公室讨价还价 | 李丽在教堂讨价还价 | 李丽在出租车上讨价还价 |
| 刘芳在自己家里母乳喂养 | 刘芳在图书馆母乳喂养 | 刘芳在公园母乳喂养 |
| 王勇在学校食堂吃煎饼 | 王勇在课堂上吃煎饼 | 王勇在公交车上吃煎饼 |
| 王涛在哲学研讨会上争辩 | 王涛在电梯里争辩 | 王涛在公园争辩 |
| 李明和女友在他家客厅拥抱 | 李明和女友在会议室拥抱 | 李明和女友在银行拥抱 |
| 张杰在酒吧里大声笑 | 张杰在葬礼上大声笑 | 张杰在公交车上大声笑 |
| 张磊在自己家里打电话 | 张磊在电影院打电话 | 张磊在邮局打电话 |
| 李霞在葬礼上大声哭 | 李霞在课堂上大声哭 | 李霞在公交车上大声哭 |
| 王军在自己房里咒骂 | 王军在参加家庭聚餐咒骂 | 王军在公园咒骂 |
| 王娟和男友在电影院手拉手 | 王娟和男友在参加商务会议手拉手 | 王娟和男友在银行手拉手 |
| 王秀兰在她家沙发上睡觉 | 王秀兰在课堂上睡觉 | 王秀兰在公园长凳上睡觉 |
| 李静在她家客厅发短信 | 李静在教堂发短信 | 李静在体育馆发短信 |
| 王芳在咖啡吧聊天 | 王芳在冥想课上聊天 | 王芳在电影院里聊天 |
| 张伟在摇滚音乐会上大喊大叫 | 张伟在图书馆大喊大叫 | 张伟在地铁上大喊大叫 |
| 王秀英在沙滩上晒日光浴 | 王秀英在葬礼上晒日光浴 | 王秀英在校园里晒日光浴 |
| 李伟在他家卧室打哈欠 | 李伟在跟老板开会打哈欠 | 李伟在听课堂讲座打哈欠 |
| 李娜在她家浴室抹口红 | 李娜在教堂抹口红 | 李娜在银行抹口红 |
| 李霞在自己家里换尿布 | 李霞在酒店大堂换尿布 | 李霞在公园换尿布 |
| 李强在参加乐队演出弹吉他 | 李强在医院里弹吉他 | 李强在地铁月台上弹吉他 |
| 刘伟在他家客厅嚼口香糖 | 刘伟在参加商务谈判嚼口香糖 | 刘伟在参加颁奖典礼上嚼口香糖 |
| 王磊在火车上听mp3 | 王磊在课堂上听mp3 | 王磊在酒吧听mp3 |
| 张丽在她家浴室里刷牙 | 张丽在酒店刷牙 | 张丽在公共厕所里刷牙 |
| 李芳在交响音乐会上鼓掌 | 李芳在葬礼上鼓掌 | 李芳在酒店大堂鼓掌 |
| 王芳在美术工作室画油画 | 王芳在医院里画油画 | 王芳在街角画油画 |
| 李梅在图书馆看书 | 李梅在教堂里看书 | 李梅在超市看书 |

*Note.* A Chinese version was used in our study.

Table S9. Items in the Sematic Violation Task Study 3 in English.

| Appropriate | Violating |
| --- | --- |
| Yang Hua crossed the gutter and mire. | Yang Hua caught the gutter and mire. |
| Wang Meng had some patulin and antipyretics. | Wang Meng rehearsed some patulin and antipyretics. |
| Wu Pin bought the Chinese brush and paper. | Wu Pin planted the Chinese brush and paper. |
| This group built the railway and bridge. | This group saved the railway and bridge. |
| Aunt Shen reapplied the phone card and id card. | Aunt Shen cleaned the phone card and id card. |
| People filled up the wallow and pond. | People planted the wallow and pond. |
| Li Jing lighted the firewood and paper scrapes. | Li Jing cursed the firewood and paper scrapes. |
| Qian Huali shot the sparrow and parrot. | Qian Huali filled in the sparrow and parrot. |
| Zheng Jun sold the sculpture and plants. | Zheng Jun hunted the sculpture and plants. |
| Wang Da broke the egg and tomato. | Wang Da expelled the egg and tomato. |
| The factory fired the ceramic and porcelain jar. | The factory sewed the ceramic and porcelain jar. |
| Constructional force built the bridge and water tower. | Constructional force opened up the bridge and water tower. |
| Wang Qiangguo performed the song and drama. | Wang Qiangguo redecorated the song and drama. |
| Xu Zheng packed a few bowls and chopsticks. | Xu Zheng freed a few bowls and chopsticks. |
| Tian Le copied the poems of dynasty tang and song. | Tian Le overwhelmed the poems of dynasty tang and song. |
| The factory smelt the iron bender and tube. | The factory sewed the iron bender and tube. |
| Uncle Li collected the dark-red enameled pottery and snuff bottle | Uncle Li performed the dark-red enameled pottery and snuff bottle |
| My father trimmed the flowers and grass. | My father closed the flowers and grass. |
| Li Dechao broke the vase and cup. | Li Dechao freed the vase and cup. |
| He Chenxiang dried the wheat and corn. | He Chenxiang cursed the wheat and corn. |
| Zhao Xiaocui sewed the socks and trouser legs. | Zhao Xiaocui froze the socks and trouser legs. |
| Zhao Ziyan browsed the newspaper and magazines. | Zhao Ziyan ate up the newspaper and magazines. |
| Secretary Sun charted the figures and tables. | Secretary Sun attracted the figures and tables. |
| Li Han poisoned the roaches and mice. | Li Han fixed the roaches and mice. |
| Wang Qing tided up the boxes and drawers. | Wang Qing harvested the boxes and drawers. |
| Wang Aihong heated the milk and soybean milk. | Wang Aihong cleaned the milk and soybean milk. |
| Grandpa Zhang expelled the ducks and swans. | Grandpa Zhang took off the ducks and swans. |
| Aunt Wang bought the candies and dessert. | Aunt Wang planted the candies and dessert. |
| They hunted the bear and pigs. | They tore the bear and pigs. |
| Yang Yi took away the keyboard and mouse. | Yang Yi copied the keyboard and mouse. |
| They killed the elephants and giraffes. | They picked the elephants and giraffes. |
| Shen Pei wiped out the sweats and tears. | Shen Pei healed the sweats and tears. |
| Wang Rongchong washed out the cabbages and onions. | Wang Rongchong tried on the cabbages and onions. |
| Liu Yang cut the cake and bread. | Liu Yang tried on the cake and bread. |
| Liu Chen stole the phone and wallet. | Liu Chen ate up the phone and wallet. |
| Yang Chao kidnapped the director and wife. | Yang Chao booked the director and wife. |
| The factory workers worked out the carpet and coat. | The factory workers forged the carpet and coat. |
| The principal told the media and public. | The principal wore the media and public. |
| Aunt Ma matched the clo thes and haircut. | Aunt Ma invited the clo thes and haircut. |
| My brother broke the glass and vase. | My brother imitated the glass and vase. |
| Wang Wei cheated the neighbors and friends. | Wang Wei segmented the neighbors and friends. |
| Ma Tao searched the corridor and the bathroom. | Ma Tao casted the corridor and the bathroom. |
| Li Ke registered the computer and luggage. | Li Ke booked the computer and luggage. |
| Kang Li fed the parrot and goldfish. | Kang Li leaked the parrot and goldfish. |
| Zhao Man followed the script and actors. | Zhao Man lobbied the script and actors. |
| Ma Wei recorded the taxes and balance. | Ma Wei acted the taxes and balance. |
| Zhang Li prepared the drinks and food. | Zhang Li registered the drinks and food. |
| Lu Rong carried the rice and plant-oil. | Lu Rong flattered the rice and plant-oil. |
| Su Mei returned the bike and key. | Su Mei answered the bike and key. |
| Li Li confirmed the property and contract. | Li Li defeated the property and contract. |

*Note.* A Chinese version was used in our study.

Table S10. Items in the Sematic Violation Task Study 3 in Chinese.

| Appropriate | Violating |
| --- | --- |
| 杨华跨过了水沟和泥潭 | 杨华捉住了水沟和泥潭 |
| 王梦雪服用了感冒药和退烧药 | 王梦雪排练了感冒药和退烧药 |
| 吴品购买了毛笔和宣纸 | 吴品栽种了毛笔和宣纸 |
| 这帮人修筑了铁路和桥梁 | 这帮人解救了铁路和桥梁 |
| 沈阿姨补办了电话卡和身份证 | 沈阿姨打扫了电话卡和身份证 |
| 人们填平了泥坑与水潭 | 人们栽种了泥坑与水潭 |
| 李靖荣点燃了木柴与纸屑 | 李靖荣痛骂了木柴与纸屑 |
| 钱华力射中了麻雀与鹦鹉 | 钱华力修平了麻雀与鹦鹉 |
| 郑军卖掉了雕塑和盆栽 | 郑军捕获了雕塑和盆栽 |
| 王达砸烂了鸡蛋和西红柿 | 王达赶跑鸡蛋和西红柿 |
| 工厂烧制出了瓷砖和瓷罐 | 工厂纺织出了瓷砖和瓷罐 |
| 工程队建造了大桥和水塔 | 工程队开垦了大桥和水塔 |
| 王强国演唱了歌曲和戏剧 | 王强国翻修了歌曲和戏剧 |
| 徐峥收拾了几只碗和筷子 | 徐峥放飞了几只碗和筷子 |
| 田乐抄写了唐诗与宋词 | 田乐压倒了唐诗与宋词 |
| 工厂冶炼出钢筋与钢管 | 工厂纺织出钢筋与钢管 |
| 李大伯收藏了紫砂壶和鼻烟壶 | 李大伯吹奏了紫砂壶和鼻烟壶 |
| 父亲修剪了花和草 | 父亲关掉了花和草 |
| 李德超打碎了花瓶和杯子 | 李德超放走了花瓶和杯子 |
| 何晨祥晾干了小麦和玉米 | 何晨祥痛骂了小麦和玉米 |
| 赵小翠缝补了袜子和裤腿 | 赵小翠冻伤了袜子和裤腿 |
| 赵紫妍浏览了报纸和杂志 | 赵紫妍吃光了报纸和杂志 |
| 孙秘书制作了图和表 | 孙秘书招来了图和表 |
| 李翰毒死了蟑螂和老鼠 | 李翰修好了蟑螂和老鼠 |
| 汪晴整理了箱子和抽屉 | 汪晴收割了箱子和抽屉 |
| 王爱红加热了牛奶和豆浆 | 王爱红清洗了牛奶和豆浆 |
| 张爷爷赶走了鸭子和天鹅 | 张爷爷摘掉了鸭子和天鹅 |
| 王阿姨购买了糖果和点心 | 王阿姨种植了糖果和点心 |
| 那伙人猎杀了熊和野猪 | 那伙人拆卸了熊和野猪 |
| 杨毅伟拿走了键盘和鼠标 | 杨毅伟抄写了键盘和鼠标 |
| 那帮人杀光了大象和长颈鹿 | 那帮人采摘了大象和长颈鹿 |
| 沈培擦掉了汗水与泪水 | 沈培治愈了汗水与泪水 |
| 王荣冲洗了白菜和大葱 | 王荣试穿了白菜和大葱 |
| 刘雅切开蛋糕和面包 | 刘雅试穿了蛋糕和面包 |
| 刘辰偷走了手机和钱包 | 刘辰吃光了手机和钱包 |
| 杨超绑架了主任及其妻子 | 杨超预订了主任及其妻子 |
| 工人编制了毛毯和大衣。 | 工人伪造了毛毯和大衣。 |
| 校长告知了媒体和公众。 | 校长穿戴了媒体和公众。 |
| 马阿姨搭配了衣服和发型。 | 马阿姨邀请了衣服和发型。 |
| 弟弟打破了玻璃和花瓶。 | 弟弟模仿了玻璃和花瓶。 |
| 王卫欺骗了邻居和朋友。 | 王卫分割了邻居和朋友。 |
| 马涛探寻了走廊和浴室。 | 马涛投掷了走廊和浴室。 |
| 李可登记了电脑和行李。 | 李可约定了电脑和行李。 |
| 康丽喂养了鹦鹉和金鱼。 | 康丽泄露了鹦鹉和金鱼。 |
| 赵曼跟随了剧本和演员。 | 赵曼游说了剧本和演员 |
| 马威记录了税金和余额。 | 马威扮演了税金和余额。 |
| 张丽准备了饮品和固体食物。 | 张丽登记了饮品和固体食物。 |
| 陆荣搬运了大米和植物油。 | 陆荣奉承了大米和植物油。 |
| 苏梅归还了自行车和钥匙。 | 苏梅回答了自行车和钥匙。 |
| 李丽核实了财产和合同。 | 李丽打败了财产和合同。 |

*Note.* A Chinese version was used in our study.


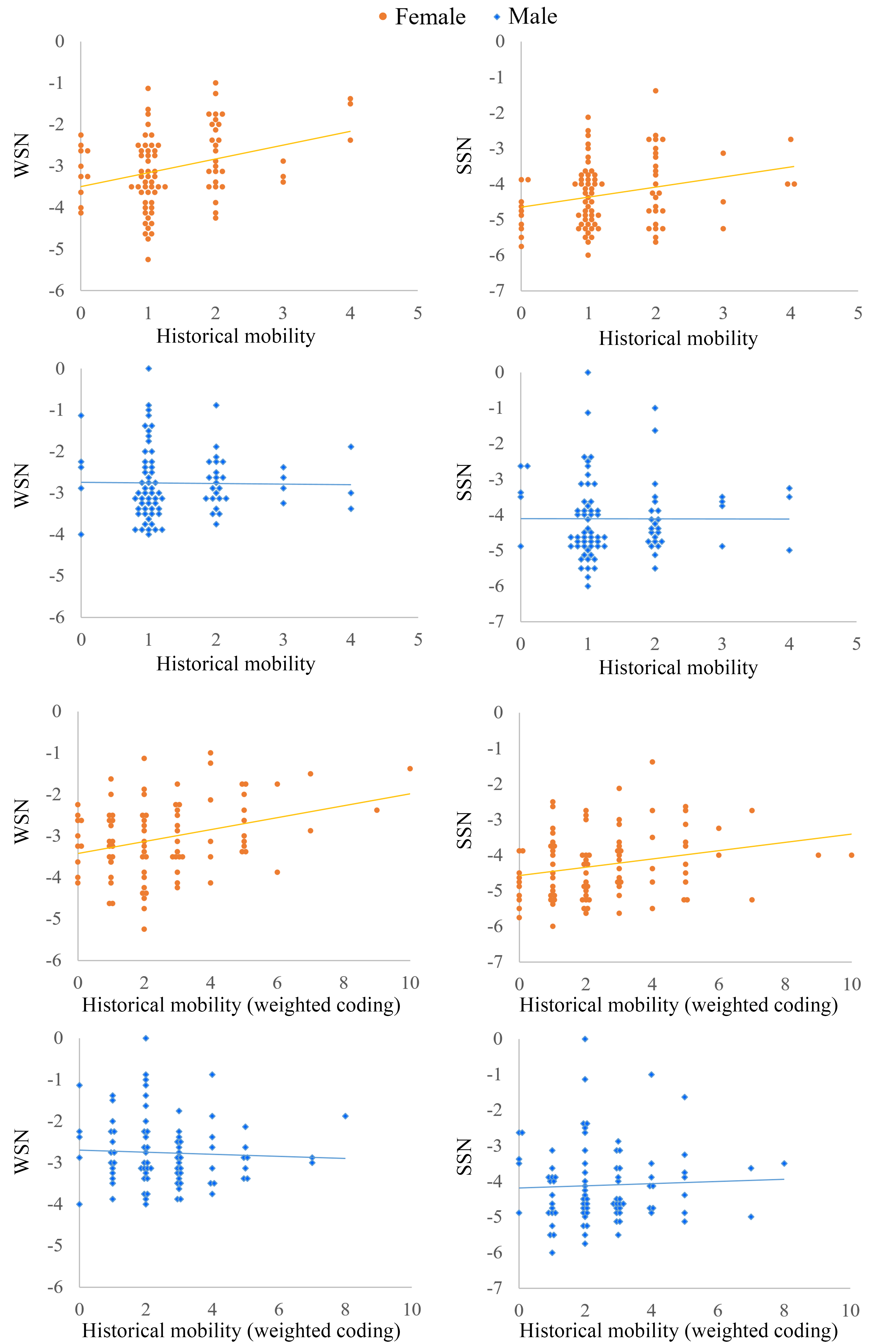


Figure S1. Association between historical mobility and WSN (original weakly inappropriate - appropriate) and SSN (original strongly inappropriate - appropriate) in two gender group. These pattern were consistent when using original historical mobility scores and weighted coding historical mobility scores (within province = 1, within nation = 2, across nation =3).


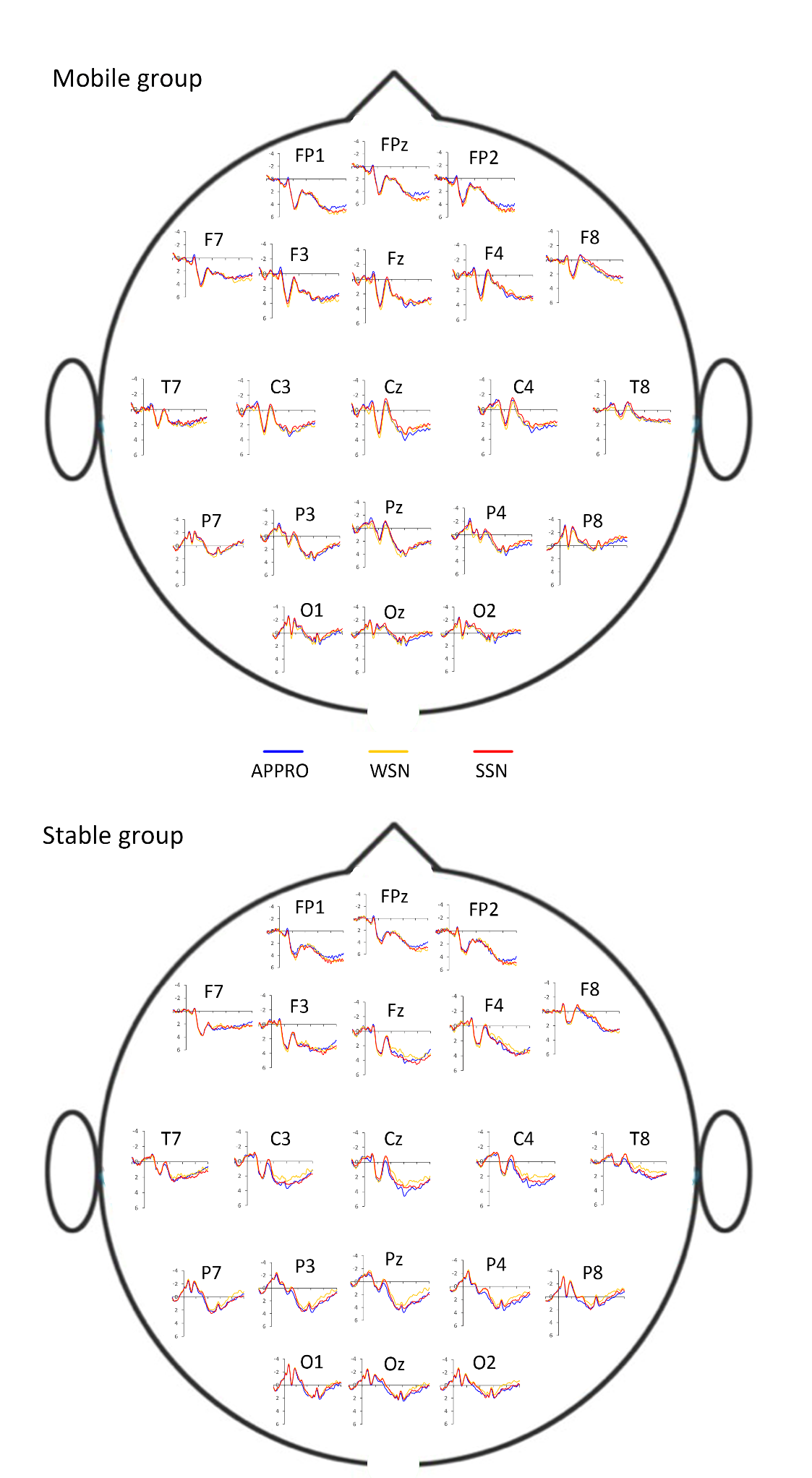


Figure S2. ERP waveforms over time in study 2.
